# Neuroprotective Effects of Trigeminal Nerve Stimulation in Severe Traumatic Brain Injury

**DOI:** 10.1038/s41598-017-07219-3

**Published:** 2017-07-28

**Authors:** Amrit Chiluwal, Raj K. Narayan, Wayne Chaung, Neal Mehan, Ping Wang, Chad E. Bouton, Eugene V. Golanov, Chunyan Li

**Affiliations:** 1 Northwell Neuromonitoring Laboratory, The Feinstein Institute for Medical Research, Manhasset, NY USA; 2 Department of Neurosurgery, Hofstra Northwell School of Medicine, Hempstead, NY USA; 3 Center for Immunology and Inflammation, The Feinstein Institute for Medical Research, Manhasset, NY USA; 4 Department of Neurosurgery, The Houston Methodist Research Institute, Houston, Texas USA; 5 Center for Bioelectronic Medicine, The Feinstein Institute for Medical Research, Manhasset, NY USA

## Abstract

Following traumatic brain injury (TBI), ischemia and hypoxia play a major role in further worsening of the damage, a process referred to as ‘secondary injury’. Protecting neurons from causative factors of secondary injury has been the guiding principle of modern TBI management. Stimulation of trigeminal nerve induces pressor response and improves cerebral blood flow (CBF) by activating the rostral ventrolateral medulla. Moreover, it causes cerebrovasodilation through the trigemino-cerebrovascular system and trigemino-parasympathetic reflex. These effects are capable of increasing cerebral perfusion, making trigeminal nerve stimulation (TNS) a promising strategy for TBI management. Here, we investigated the use of electrical TNS for improving CBF and brain oxygen tension (PbrO_2_), with the goal of decreasing secondary injury. Severe TBI was produced using controlled cortical impact (CCI) in a rat model, and TNS treatment was delivered for the first hour after CCI. In comparison to TBI group, TBI animals with TNS treatment demonstrated significantly increased systemic blood pressure, CBF and PbrO_2_ at the hyperacute phase of TBI. Furthermore, rats in TNS-treatment group showed significantly reduced brain edema, blood-brain barrier disruption, lesion volume, and brain cortical levels of TNF-α and IL-6. These data provide strong early evidence that TNS could be an effective neuroprotective strategy.

## Introduction

Traumatic brain injury (TBI) is a leading cause of mortality and morbidity worldwide and has a significant socioeconomic impact^1, 2^. In particular, TBI represents a third of all injury related deaths, and is associated with significant disability among survivors. Despite significant advances in our understanding of the pathophysiology and management of this serious condition, to date, there are no drugs that have shown to improve patient outcomes significantly. Therefore, there is a clear need for developing novel therapeutic strategies to maximize recovery after TBI.

TBI is classified as either primary or secondary^1, 3–6^. The immediate mechanical destruction of the tissue and blood vessels that happens at the time of impact is the primary injury, and the damage is irreversible. Secondary injury develops in a gradual fashion in response to the mechanical damage, and is prompted by a multitude of factors, with ischemia playing a central role^4, 6, 7^. In the critical early hours after trauma, the perilesional area becomes hypermetabolic, but at the same time there is a decrease in cerebral blood flow (CBF)^8–13^. This mismatch of supply and demand leads to an energy crisis at the cellular level, resulting in additional death of neural tissue in the ischemic penumbra. This process of secondary brain injury leads to further decline in neurological function and poor outcome. Therefore, early augmentation of blood flow to the brain, especially in the perilesional area is essential in preventing secondary insult^14^. In fact, the prevention of secondary brain injury has been the major focus of neurotrauma research and clinical practice; however treatment options have been limited to blood pressure and intracranial pressure management.

Neuromodulation is a therapeutic technique that involves modification of neural function via external stimulation, usually in the form of electrical impulses. Prominent examples of neuromodulative therapies are vagus nerve stimulation for epilepsy and deep brain stimulation for Parkinson’s Disease^15, 16^. Trigeminal nerve stimulation (TNS) has also been an active area of research in a variety of disease processes, and has shown efficacy in the treatment of epilepsy and depression^17, 18^. Its role in other pathological conditions such as posttraumatic stress disorder (PTSD), trigeminal neuralgia, and migraine headaches is ongoing^19–21^.

**Figure 1:**
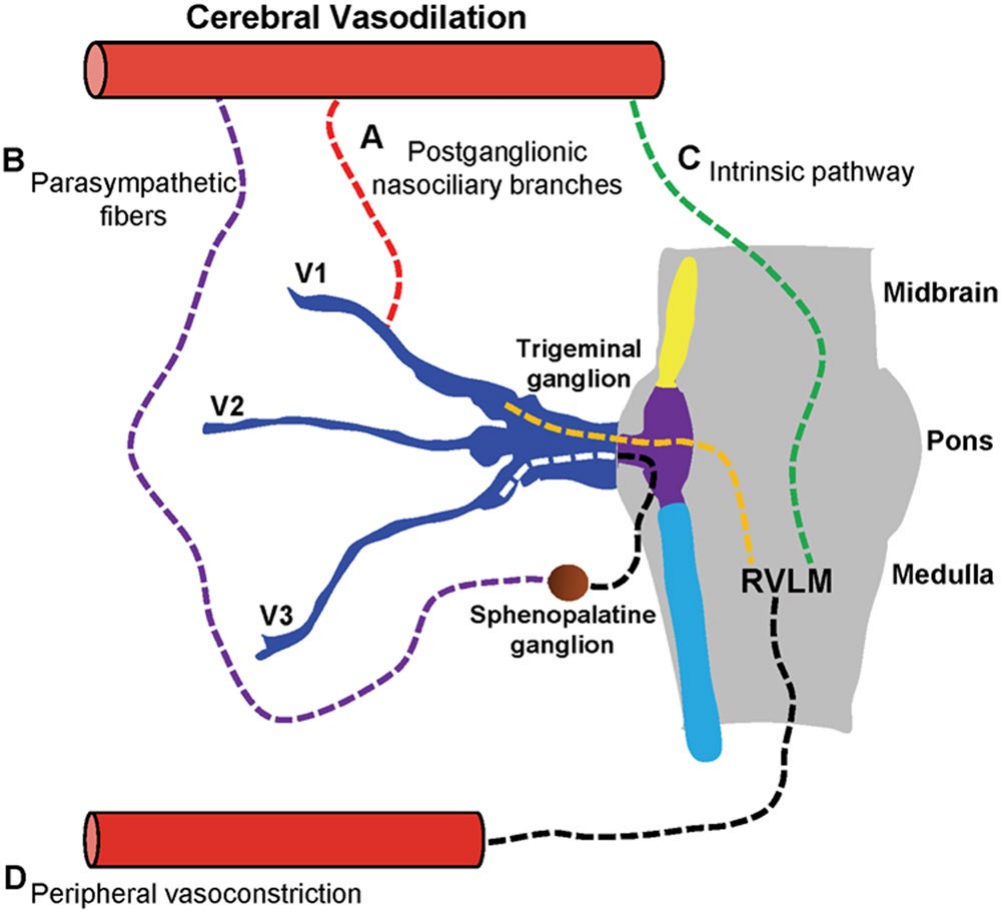
Schematic representation of the neuroanatomical projections of trigeminal nerve for increased cerebral blood flow (CBF). (**A**) Postganglionic sensory fibers from V1 (ophthalmic nerve) innervates cerebral vessels and causes vasodilation via axon-reflex mechanism. (**B**) Trigemino-parasympathetic reflex arc involved in vasodilation. (**C**) Activation of rostral ventrolateral medullar (RVLM) causes cerebrovasodilation via intrinsic pathway. (**D**) Increased mean arterial pressure (MAP) via RVLM-sympathetic pathway leads to increased CBF.

The trigeminal nerve (Cranial Nerve V) is the largest cranial nerve, offering a high-bandwidth pathway for signals to enter the brain bilaterally and at high frequency. It projects directly or indirectly to several areas of the brain, such as the locus coeruleus, nucleus tractus solitaries (NTS), rostral ventrolateral medullar (RVLM), thalamus and the cerebral cortex^21, 22^. There are two important properties of the trigeminal nerve that make it a potentially viable target in TBI management. Stimulation of the trigeminal nerve causes a vasopressor response by activating the RVLM, an important brainstem vasomotor center^23, 24^. Furthermore, TNS has been shown to decrease cerebrovascular resistance via the trigemino-cerebrovascular system^25^. When activated, these pathways, via RVLM and trigemino-cerebrovascular system, can lead to significant increase in cerebral perfusion, hence making the trigeminal nerve a promising target in TBI management (Fig. 1). However, to date TNS has not been investigated for the treatment and prevention of secondary brain injury after TBI.

In this study, we hypothesized that TNS can decrease secondary damage after severe TBI by increasing CBF and delivering more oxygen to the injured brain. Using a rat controlled cortical impact (CCI) model, we demonstrated that intermittent electrical TNS delivered immediately after CCI for 60 minutes (1-minute stimulation for every 11 minutes interval) significantly improved CBF and brain tissue oxygen levels. Moreover, we showed that TNS was capable of decreasing brain edema, blood-brain barrier (BBB) disruption, lesion volume, and inflammatory response as evaluated by the levels of TNF-α and IL-6 in brain tissue.

## Results

### Effects of Direct TNS on Hemodynamics in Naïve Rats

**Figure 2:**
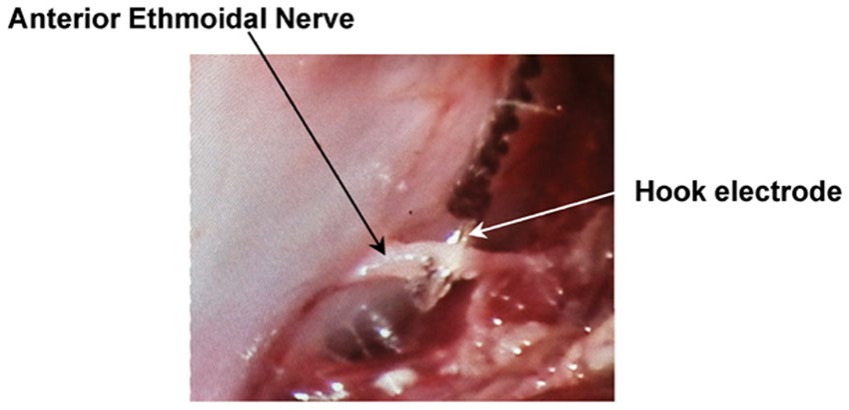
Direct trigeminal nerve stimulation (TNS). Anterior Ethmoidal nerve (AEN) has been isolated, and hook electrode has been directly placed on the right and left branches of AEN to provide bilateral stimulation.

**Figure 3:**
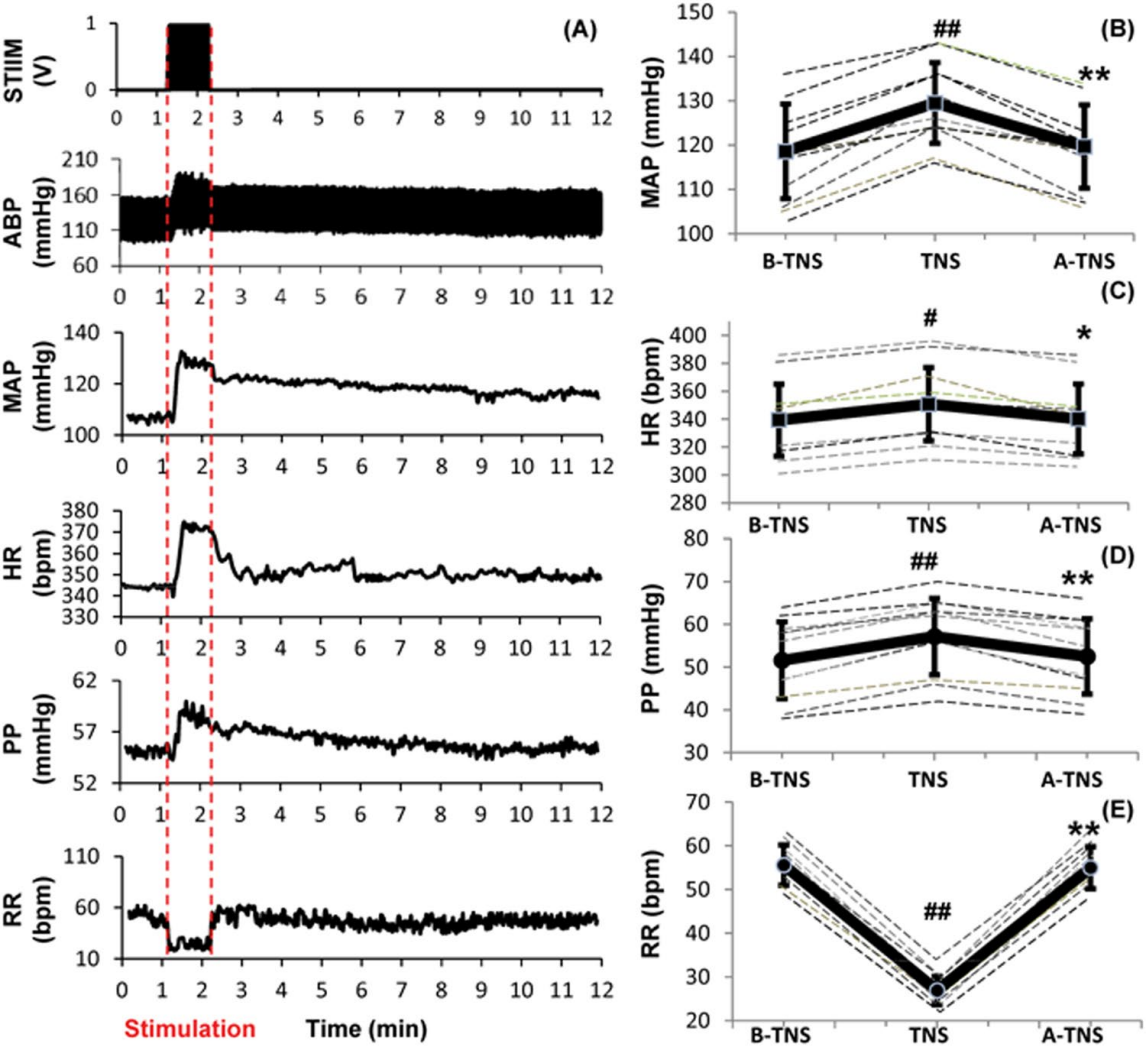
Effects of direct trigeminal nerve stimulation (TNS) on hemodynamics. (**A**) Representative changes in MAP, HR, PP and RR. (**B**) MAP increased significantly with TNS. (**C**) HR increased slightly with TNS. (**D**) PP increased with TNS. (**E**) RR decreased significantly. Data were expressed as mean ± SD and analyzed using repeated measures ANOVA. ^#^p < 0.05 vs baseline, ^##^p < 0.001 vs baseline, ^*^p < 0.05 vs TNS, ^**^p < 0.001 vs TNS, n = 12/group. B-TNS: 1-min before trigeminal nerve stimulation; TNS: during 1-min trigeminal nerve stimulation; A-TNS: 10-min after trigeminal nerve stimulation.

We examined the effects of direct trigeminal nerve branch stimulation (the right and left anterior ethmoidal nerves as shown in Fig. 2) on mean arterial blood pressure (MAP), pulse pressure (PP), heart rate (HR), and respiration rate (RR) in naïve rats. One direct TNS were delivered to each rat after 30-min baseline recording. One of the representative recordings is shown in Fig. 3A. During the 1-minute direct TNS, MAP significantly increased by 9.2% compared to the baseline (129.5 ± 9.1 vs. 118.6 ± 10.7 mmHg; p < 0.001; n = 12) (Fig. 3B). PP and HR increased from 52 ± 9 to 57 ± 9 mmHg (p < 0.001 n = 12), and 340 ± 26 to 351 ± 26 bpm (p < 0.05 n = 12), respectively (Fig. 3C and D). RR decreased by 52% compared to the baseline (27 ± 3 vs. 56 ± 5 bpm; p < 0.001; n = 12) concurrently (Fig. 3E). All the values returned to their baseline within 10 minutes.

### Effects of Subcutaneous TNS on Hemodynamics in Naive Rats

**Figure 4:**
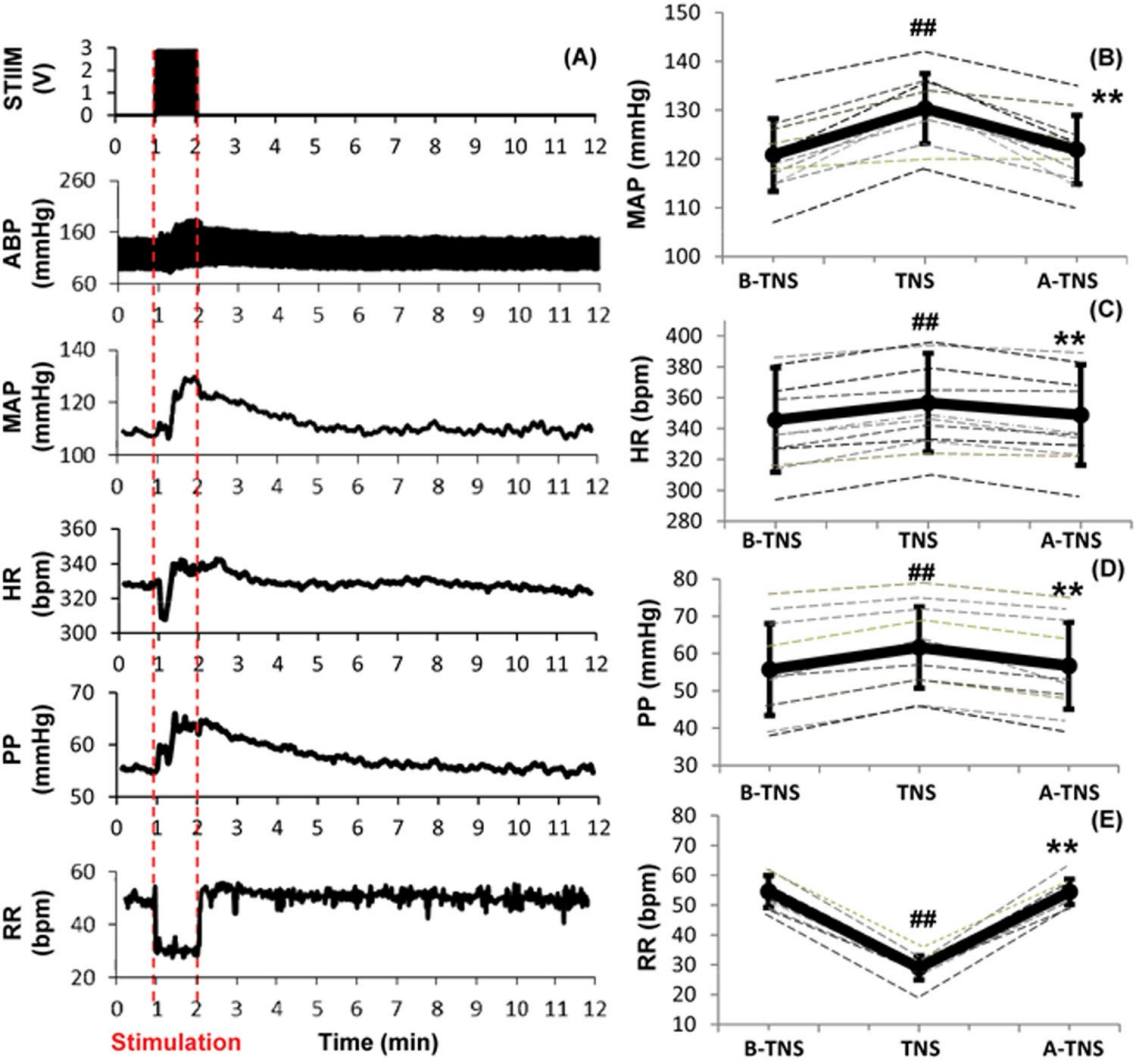
Effects of subcutaneous trigeminal nerve stimulation (TNS) on hemodynamics. (**A**) Representative changes in MAP, HR, PP and RR. (**B**) MAP increased significantly with TNS. (**C**) HR increased slightly with TNS. (**D**) PP increased with TNS. (**E**) RR decreased significantly. Data were expressed as mean ± SD and analyzed using repeated measures ANOVA. ^#^p < 0.05 vs baseline, ^##^p < 0.001 vs baseline, ^*^p < 0.05 vs TNS, ^**^p < 0.001 vs TNS, n = 12/group. B-TNS: 1-min before trigeminal nerve stimulation; TNS: during 1-min trigeminal nerve stimulation; A-TNS: 10-min after trigeminal nerve stimulation.

In order to minimize the invasive procedures of direct TNS, we stimulated the terminal branches of trigeminal nerve via minimally-invasive subcutaneous electrodes in the distribution of V1 (ophthalmic branch). Stimulation parameters were tuned so that subcutaneous TNS results in similar hemodynamic changes as direct TNS. One subcutaneous TNS were delivered to each rat after 30-min baseline recording. Figure 4A shows one of representative recordings. During the 1-minute direct TNS, MAP increased by 8.1% compared to the baseline (130.6 ± 6.7 vs. 120.8 ± 7.5 mmHg; p < 0.001; p = 12), and gradually returned back to baseline within 10 minutes (Fig. 4B). PP and HR increased from 56 ± 12 to 62 ± 11 mmHg (p < 0.001 n = 12), and 346 ± 34 to 357 ± 32 bpm (p < 0.001 n = 12), respectively (Fig. 4C and D). RR significantly decreased by 46.9% compared to the baseline (29 ± 4 vs. 55 ± 5 bpm; p < 0.001; n = 12) (Fig. 4E).

### Effects of Subcutaneous TNS on Cerebral Parameters in Naïve Rats

**Figure 5:**
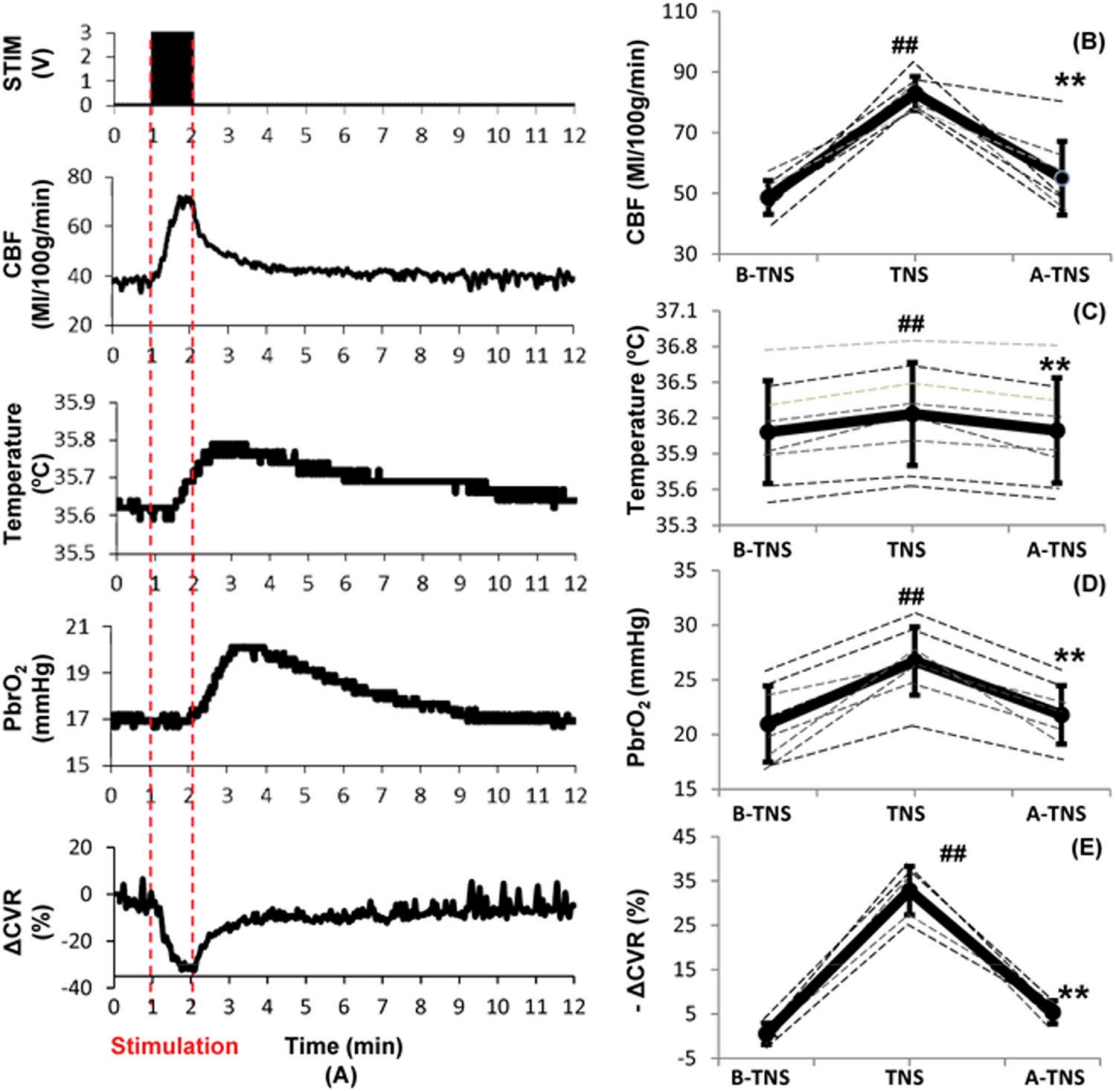
Effects of subcutaneous trigeminal nerve stimulation (TNS) on cerebral parameters. (**A**) Representative changes in CBF, brain temperature, PbrO_2_ and ΔCVR. (**B**) CBF increased significantly with TNS. (**C**) Brain temperature increased slightly with TNS. (**C**) PbrO_2_ increased significantly with TNS. (**D**) ΔCVR decreased significantly with TNS. Data were expressed as mean ± SD and analyzed using repeated measures ANOVA. ^#^p < 0.05 vs baseline, ^##^p < 0.001 vs baseline, ^*^p < 0.05 vs TNS, ^**^p < 0.001 vs TNS, n = 6~8/group. B-TNS: 1-min before trigeminal nerve stimulation; TNS: during 1-min trigeminal nerve stimulation; A-TNS: 10-min after trigeminal nerve stimulation.

1-minute application of subcutaneous TNS triggered significant changes in cerebral parameters. Representative recordings of CBF, PbrO_2_, brain temperature and cerebrovascular resistance (CVR) are shown in Fig. 5A. CBF increased from 48.7 ± 5.6 to 82.9 ± 5.6 ml/100 g/min (p =< 0.001; n = 8), a 70.4% increase compared to the baseline which slowly decreased to baseline in 10 minutes (Fig. 5B). There was a lag of approximately 51 ± 33 sec until PbrO_2_ started to increase by 5% over its baseline. From the baseline value of 20.9 ± 3.5 mmHg, it increased to 26.7 ± 3.1 mmHg (p < 0.001; n = 8), a 27.4% increase, and again gradually decreased to baseline in 10 minutes (Fig. 5C). TNS caused a 0.4% increase in cerebral temperature from 36.08 ± 0.42 °C to 36.23 ± 0.43 °C (p < 0.001; n = 8). There was a lag of approximately 26 ± 16 sec until the temperature started to increase by 5% over its baseline and after stimulation it kept increased for 15 ± 9 sec until it reached its maximum (Fig. 5D). CVR was calculated as a ratio between MAP and CBF and expressed as percentage of change relative to the baseline. In parallel with CBF increase, CVR decreased to a minimum of 32.8% (p =< 0.001; n = 6) gradually returning to the baseline in 10 minutes (Fig. 5E).

### Effects of CCI on Hemodynamics and Cerebral Parameters

**Figure 6:**
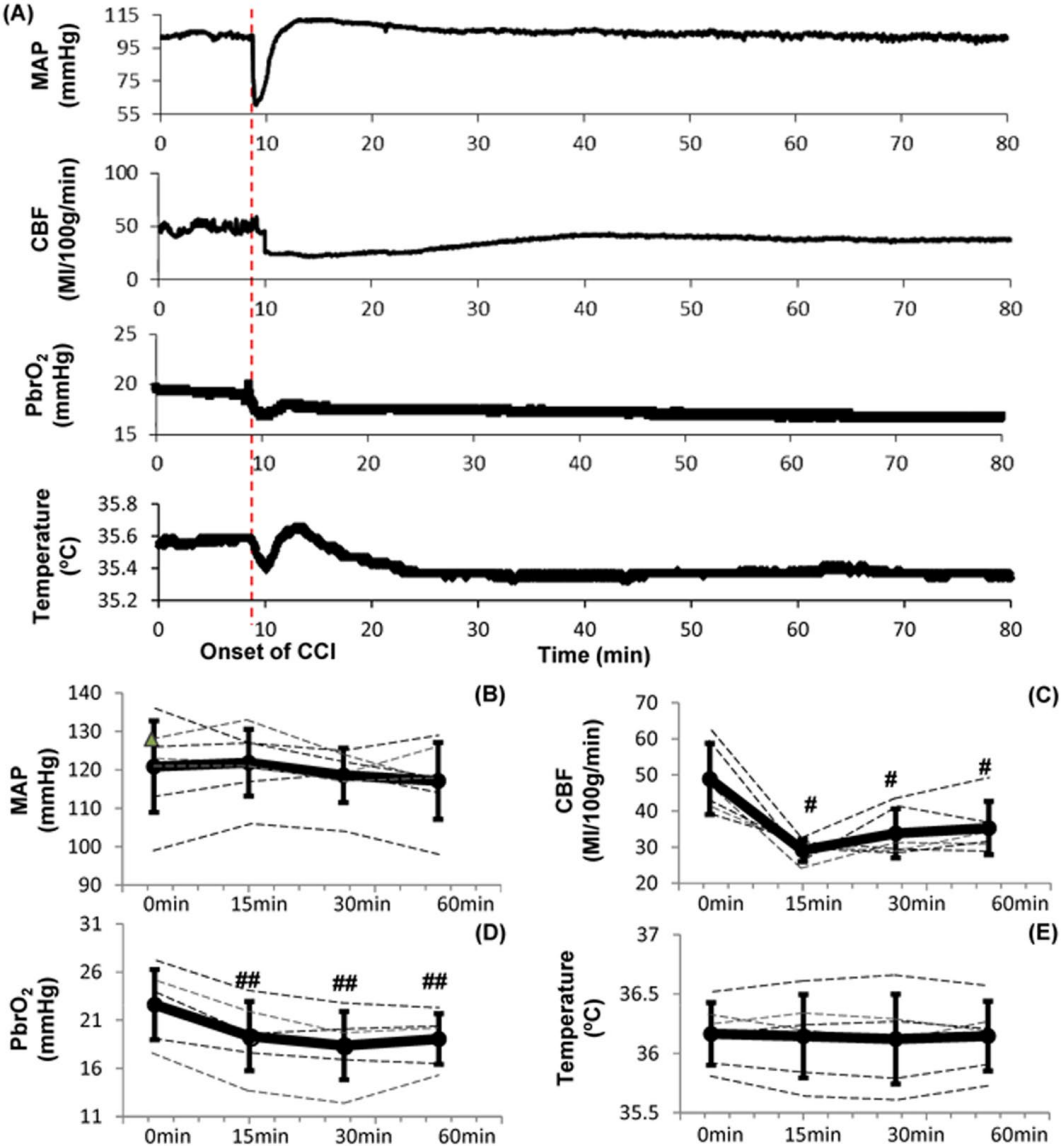
Effects of controlled cortical impact (CCI) on hemodynamics and brain function. (**A**) Representative changes in MAP, CBF, PbrO_2_ and brain temperature. (**B**) MAP decreased significantly immediately after CCI. However, no significant changes on MAP at 15 min, 30 min and 60 min after CCI. (**C**) CBF decreased significantly at 15 min, 30 min and 60 min after CCI. (**D**) PbrO_2_ decreased significantly at 30 min and 60 min after CCI. (**E**) No significant changes on brain temperature after CCI. Data were expressed as mean ± SD and analyzed using repeated measures ANOVA. ^#^p < 0.05 vs baseline, ^##^p < 0.001 vs baseline, n = 6~7/group.

Representative changes in hemodynamics and cerebral parameters after CCI-induced severe TBI are shown in Fig. 6A. At the onset of CCI, there was a rapid decrease in MAP, CBF, and PbrO_2_. MAP rapidly decreased to a minimum of 27.9% compared to the baseline (87.17 ± 10.83 vs. 120.83 ± 13.04; p =< 0.001; n = 6) in 1 minute and started to increase returning to the baseline approximately in 2 minutes. Within 15 minutes, MAP and temperature recovered to their baseline (Fig. 6B). CBF and PbrO_2_ however remained decreased at 15, 30 and 60 minutes. Compared to its baseline value of 49.4 ± 9.1 ml/100 g/min, CBF at 15, 30 and 60 minutes after CCI were 30.9 ± 5.5 (p < 0.05; n = 7), 34.3 ± 6.3 (p < 0.05; n = 7), 35.1 ± 6.8 ml/100 g/min (p < 0.05; n = 7), 37.4%, 30.5%, 29% decline, respectively (Fig. 6C). With CBF decrease, PbrO_2_ continuously dropped by 14.3% compared to the baseline (19.17 ± 3.30 vs. 22.37 ± 3.39 mmHg; p < 0.001; n = 7) at 15 minutes, followed by a less steep but prolonged further decrease by 17.9% (18.35 ± 3.22 vs. 22.37 ± 3.39 mmHg; p < 0.001; n = 7) and 16.2% (18.74 ± 2.42 vs. 22.37 ± 3.39 mmHg; p < 0.001; n = 7) at 30 and 60 minutes respectively (Fig. 6D). Brain temperature depends on the bleeding status after CCI. If there was no bleeding after CCI, the cortical temperature increased by 0.007 ± 0.002% (p < 0.001; n = 6) approximately in 2 minutes. If there was bleeding, the cortical temperature first decreased, and then either slightly increased by 0.002% or kept decreasing (Fig. 6E).

### Effects of Subcutaneous TNS on CCI-induced TBI

**Figure 7:**
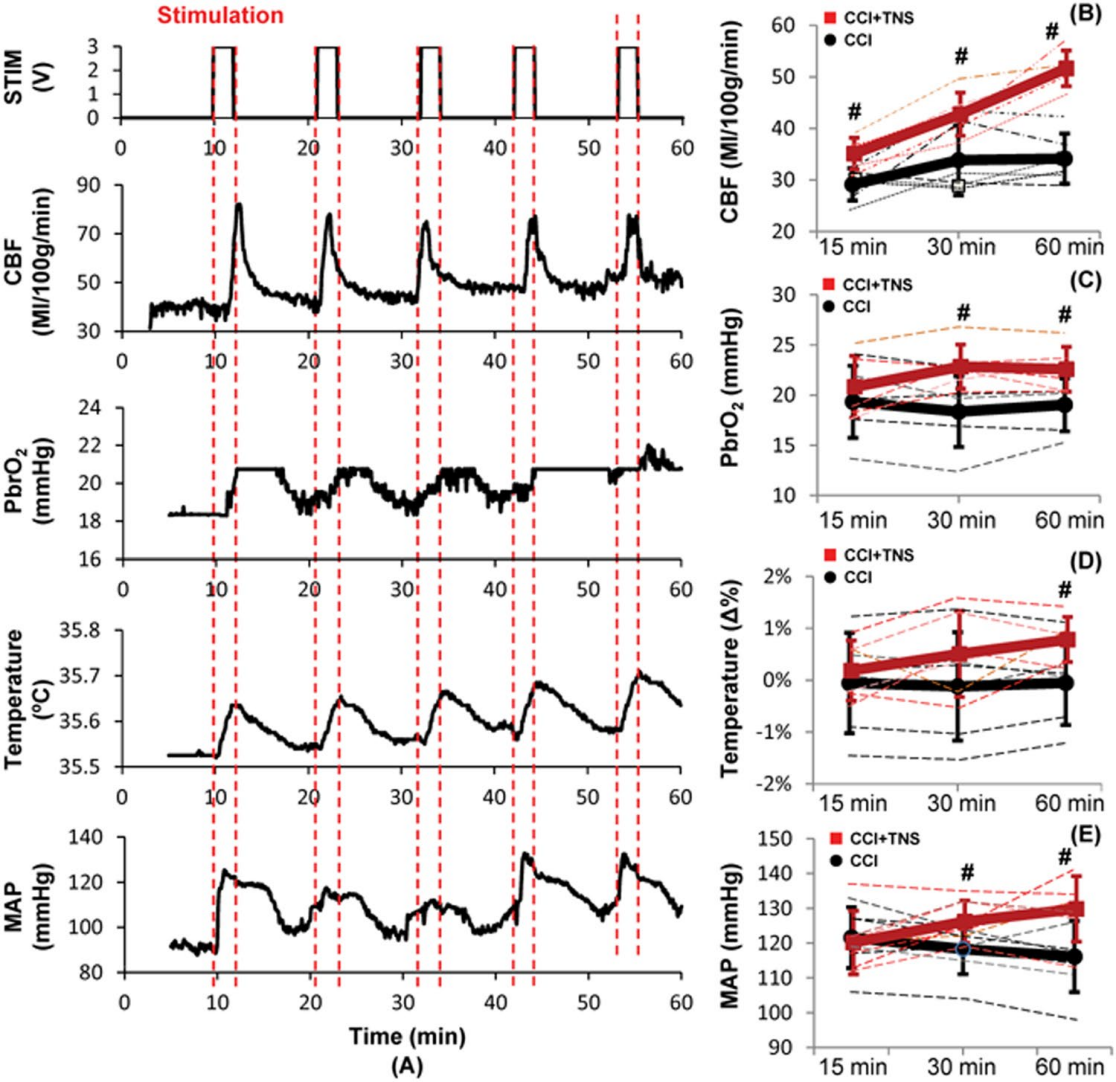
Effects of subcutaneous TNS on CCI-induced TBI. (**A**) Representative changes in CBF, PbrO_2_, brain temperature and MAP with intermittent TNS. (**B**) CBF increased significantly at 15 min, 30 min and 60 min after CCI. (**C**) PbrO_2_ increased significantly at 30 min and 60 min after CCI. (**D**) Brain temperature changes increased significantly at 60 min after CCI. (**E**) MAP increased significantly at 30 min and 60 min after CCI. Data were expressed as mean ± SD and analyzed using student’s t test. ^#^p < 0.05 vs CCI-induced TBI, n = 6~7/group. Red: TBI + TNS; Black: TBI.

At 10 minutes after the CCI, 1-minute TNS in every 11 minutes was delivered for 60 minutes (total 5 TNS). Figure 7A shows a representative recording of the changes in CBF, PbrO_2_, brain temperature and MAP after CCI following by TNS. CBF, PbrO_2_ and MAP showed marked improvement. At 15, 30, and 60 minutes after the CCI, the mean CBF of the TNS-treated rats was 35.1 ± 3.1, 42.8 ± 4.2, and 51.7 ± 3.5 ml/100 g/min, 20.5% (p = 0.007; n = 6), 26.4% (p = 0.0205; n = 6) and 51.3% (p =< 0.001; n = 6) increase compared to the TBI rats, respectively (Fig. 7B). The PbrO_2_ values at 15, 30, and 60 minutes for the TNS-treated rats were 20.8 ± 3.1, 22.9 ± 2.2, and 22.6 ± 2.2 mmHg, 7.7% (p = 0.23; n = 6), 24.4% (p = 0.012; n = 6), and 18.5% (p = 0.015; n = 6) increase compared to the TBI rats, respectively (Fig. 7C). There was no statistical difference in the brain temperature changes (=[(t_x-min_ − t_0-min_)/t_0-min_] ×100%) between the 2 groups at 15 and 30 minutes after the CCI (Fig. 7D). However, brain temperature changes showed significant increase in the TNS-treated group at 60 minutes compared to the TBI rats (−0.051 ± 0.8% vs. 0.79 ± 0.43%; p = 0.0252; n = 6). MAP was also significantly higher in the TNS-treated group at 30 minutes (127.0 ± 6.0 vs. 118.3 ± 7.2 mmHg) and 60 minutes (129.7 ± 8.6 vs. 116.1 ± 10.2 mmHg) compared to the TBI rats (119.0 ± 12.9 mmHg), 7.4% (p = 0.015; n = 7) and 11.7% (p = 0.01; n = 7) increase, respectively (Fig. 7E).

### Effects of Subcutaneous TNS on Brain Damage

**Figure 8:**
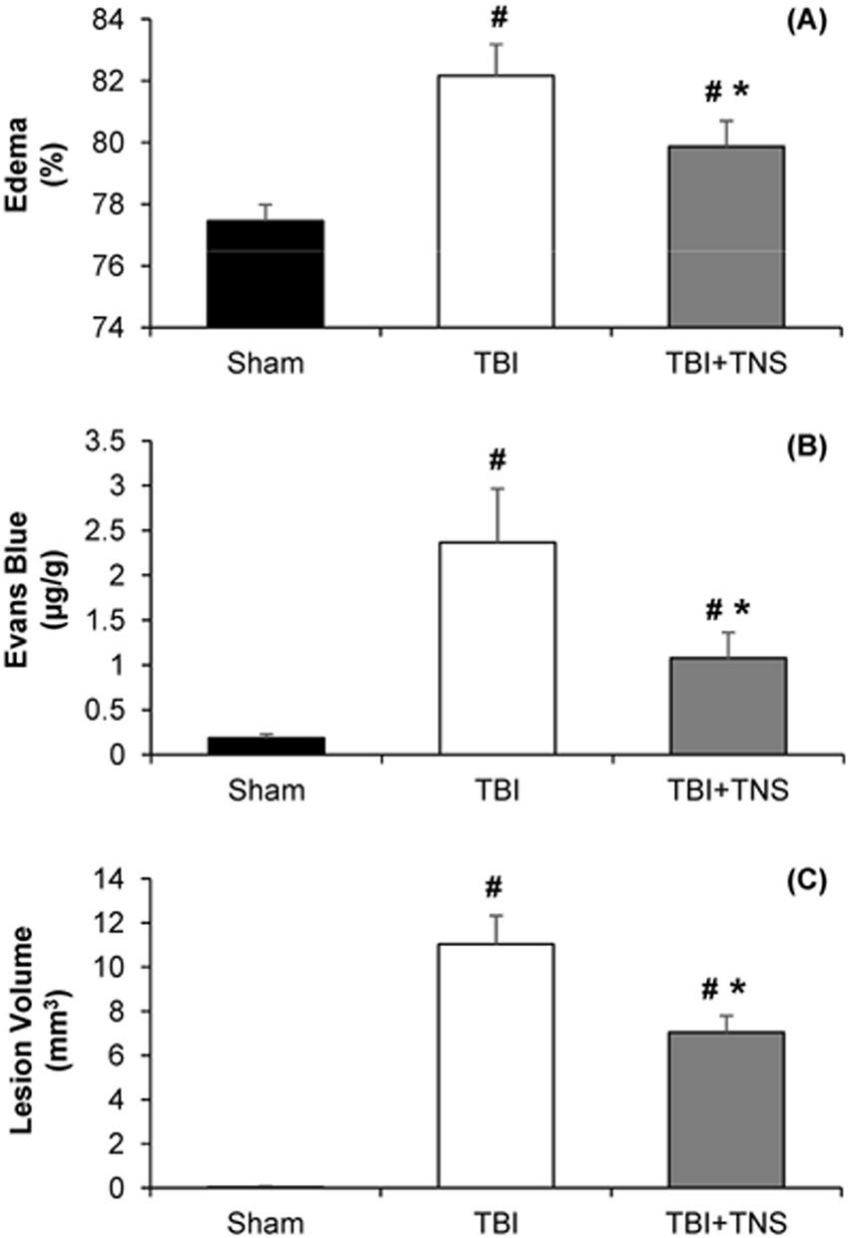
Effects of subcutaneous TNS on brain damage. (**A**) Brain edema decreased significantly at 24 h after CCI-induced severe TBI. (**B**) TNS ameliorated blood-brain barrier (BBB) disruption. (**C**) Lesion volume decreased significantly with TNS treatment. Data were expressed as mean ± SD and compared by one-way ANOVA and Student-Newman-Keuls test. ^#^p < 0.05 vs sham, ^*^p < 0.05 vs CCI-induced TBI, n = 6/group.

CCI caused significant increase in brain edema, BBB disruption, and lesion volume at 24 hours post-injury. Animals that received CCI had significant increase in edema level compared to the sham-control groups (82.2 ± 1.0 vs. 77.5 ± 0.5%; p =< 0.001; n = 6). Mean edema level for TNS-treatment animals was 79.9 ± 0.8%, 6.1% decrease (p =< 0.001; n = 6) compared to TBI animals (Fig. 8A). CCI also significantly disrupted the BBB compared to sham groups (2.37 ± 0.60 vs. 0.19 ± 0.04 µg/g; p = 0.002; n = 6). Evans blue dye concentration in TNS-treatment group was 1.08 ± 0.28 µg/g, which is a 54.4% decrease (p =< 0.001; n = 6) compared to TBI-control animals (Fig. 8B). Mean lesion volume for TNS-treatment rats was 7.04 ± 0.75 mm^3^, 36.1% decrease (p < 0.001; n = 6) compared to the TBI rats (11.03 ± 1.29 mm^3^) (Fig. 8C).

### Effects of Subcutaneous TNS on Inflammation

**Figure 9:**
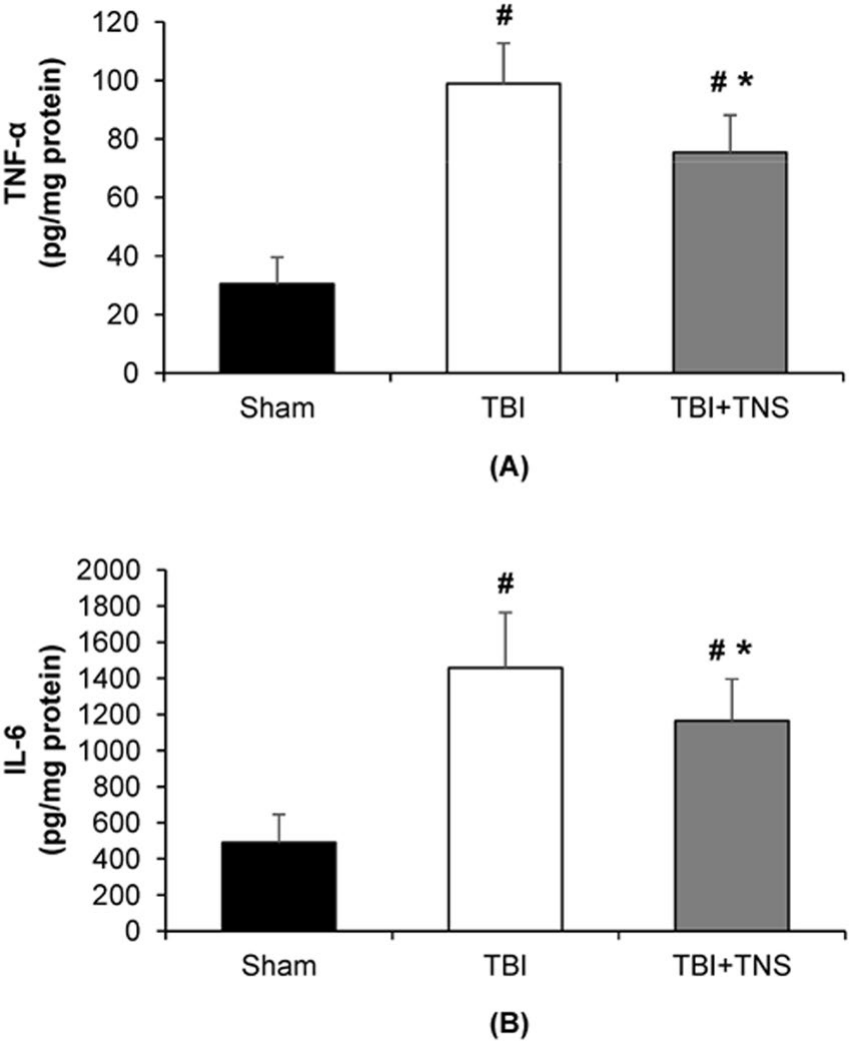
Effects of subcutaneous TNS on brain inflammation. (**A**) Brain levels of TNF-α. (**B**) Brain levels of IL-6 decreased significantly at 4 h after CCI-induced severe TBI. Data were expressed as mean ± SD and compared by one-way ANOVA and Student-Newman-Keuls test. ^#^p < 0.05 vs sham, ^*^p < 0.05 vs CCI-induced TBI, n = 8/group.

TBI initiates a cascade of inflammatory processes that can serve to exacerbate the initial injury. As shown in Fig. 9, brain cortical tissue levels of TNF-α and IL-6 increased significantly by 225% and 197% at 4 h after TBI. TNS treatment decreased brain cortical levels of TNF-α and IL-6 by 23.8% (75.4 ± 12.7 vs. 98.9 ± 13.9 pg/mg protein; p = 0.003; n = 8) and 20.1% (1165.3 ± 231.1 vs. 1458.3 ± 305.9 pg/mg protein; p = 0.048; n = 8) compared to the TBI group, respectively.

## Discussion

Ischemia plays a central role in the pathophysiology of secondary brain injury in the early post TBI period. Therefore, in this study we investigated whether augmenting blood flow to the brain via TNS can attenuate injury progression. Previous studies have shown that brain tissue around the injured area are prone to ischemia, either due to compression from the adjacent traumatic mass lesions or due to hypermetabolic activities from the initiation of biochemical, physiological and electrical cascades^26–28^. After the primary insult, damaged neurons, astrocytes and microglia release reactive oxygen species, excitatory neurotransmitters and pro-inflammatory cytokines^1^. Initiation of inflammatory cascades lead to further breakdown of the blood brain barrier, causing edema and intracranial hypertension. These changes in the cerebral microenvironment in and around the damaged tissue occur in a gradual fashion, starting from the time of the primary injury and continuing for days. There is also a reduction of blood flow to the pericontusional area after the initial insult by as much as 50% compared to normal levels^8^. This combination of hypermetabolism and reduction in CBF make the perilesional penumbra exquisitely vulnerable to ischemic damage. There is also evidence that ischemia itself induces production of pro-inflammatory cytokines and triggers inflammatory cascades^29, 30^. In our animal model, we were able to replicate these previously described indicators of secondary damage after severe TBI. CCI caused a sustained decrease in cerebral blood flow and oxygen tension in the penumbra of the lesion as the animals were being monitored at the hyperacute phase of TBI. Furthermore, there were increases in the markers of neuroinflammation at 4 hours and in cerebral edema, breakdown of blood brain barrier, and lesion volume at 24 hours post-injury.

In contrast, animals exposed to TNS demonstrated attenuation of these pathological changes. After the initiation of TNS, there was an instantaneous elevation of MAP, and at 60 minutes after the CCI-induced TBI, MAP was 11.7% higher in the treatment rats. Similar trends were observed with CBF and PbrO_2_ with 51.3% and 18.5% higher values, respectively in the treatment group. In current clinical practice, elevating MAP using intravenous fluids or vasoactive agents is one of the principle tools in TBI management, as it improves cerebral perfusion. As such, AANS-Brain Trauma Foundation guidelines recommend maintaining systolic blood pressure (SBP) of at least 100 mmHg in patients with severe TBI in order to minimize the chances of secondary brain injury^14^.

In this study, we were able to show that blood pressure can be elevated with TNS by exploiting existing neural pathways via the trigeminal nerve. Viral tracing studies of ethmoidal nerve have shown that these fibers project to multiple brainstem nuclei, including RVLM, nucleus of the tractus solitarius (NTS), lateral tegmental field, superior salivatory nucleus (SSN) and Kolliker Fuse nucleus (KFN)^31–33^. Of these, the RVLM is most closely associated with the systemic vasomotor control. It contains C1 group of cells that are adrenergic, and they play a pivotal role in maintaining systemic vascular tone via modulation of intermediolateral column cells and sympathetic output^22, 33, 34^. TNS activates these C1 cells, which ultimately results in peripheral vascular resistance^35, 36^. With peripheral vasoconstriction, blood is shunted centrally and directed towards more ischemia prone organs, such as the heart and the brain.

One important observation in our study is that while MAP increased by 8.1%, CBF increased by 70.4% in the naive rats. Similarly, in TNS-treated rats, MAP increased by 11.7% and CBF increased by 51.3% compared to TBI-control rats. Therefore, it is reasonable to assume the increase in CBF is not just due to increased MAP. This is corroborated by the fact that CVR in the naive rats decreased by 32.8% during stimulation, which indicates that there was active cerebral vasodilation with TNS. This observation is consistent with multiple previous studies in both animals and humans^37–43^. The distal trigeminal nerve branches richly innervate cerebral vasculature, and this entity is often referred to as the trigemino-cerebrovascular system^25^. The exact mechanism of how activation of the trigeminal nerve causes cerebral vasodilation is not fully understood, but there is evidence that the effect is primarily mediated by postganglionic trigeminal fibers, principally V1 branches^44, 45^. In animal studies, it has been shown that postganglionic trigeminal fibers play a role in attenuating vasospasm after SAH, and that lower density of V1 fibers in the vasculature have been associated with increased incidence of spasm, which is highly suggestive of the trigeminal nerve having tonic control of cerebral vessel diameter^46–48^. These fibers contain calcitonin gene-related peptide (CGRP) and Substance P at their synaptic terminals, and when stimulated, these potent vasodilatory substances are released, resulting in vasodilation^41, 49, 50^.

There is also evidence that the vasodilatory effect of the trigeminal nerve is mediated in part by the parasympathetic fibers from the sphenopalatine ganglion, with acetylcholine and vasoactive intestinal polypeptide (VIP) as mediators^51–53^. This pathway involves primarily V3 (mandibular nerve) as the afferent arm, and parasympathetic fibers via the sphenopalatine or otic ganglion as the efferent arm^51^. It should be noted that this pathway was not specifically targeted in this study. Furthermore, RVLM itself has been shown to cause cerebral vasodilation independent of changes in cerebral oxygen consumption (CMRO_2_) or cerebral glucose utilization (CMRGlc)^22, 54, 55^. All of the different ways that TNS can increase CBF has been summarized in Fig. 1. While this study was not specifically designed to determine the relative contribution of increase in CBF from each of these pathways, it is reasonable to postulate that the effect was mostly due to the activation of trigemino-cerebrovascular system via the distal V1 branches, as the needle electrodes were placed in the V1 distribution.

Enlargement of lesion volume beyond the initial impact size, increased edema and BBB permeability are key indicators of secondary brain injury. With improvement in perfusion, our results show that there were significant decreases in edema level, BBB permeability and lesion volume. The average edema level, the extent of BBB permeability, and lesion volume in the TNS-treatment group were 6.1%, 54.4% and 36.1% less than the TBI-control group, respectively. We attribute this difference to the improvements in CBF and PbrO_2_ secondary to TNS treatment. With blood flow augmentation, it is conceivable to think that at the cellular level, both ischemia and hypoxia, the two key contributors of secondary damage, are being reduced in the penumbra, which resulted in the salvage of vulnerable neuronal tissue.

Besides ischemia and hypoxia, neuroinflammation is another major contributor to secondary brain injury. In fact, the use of anti-inflammatory agents, such as minocycline and peroxisome proliferator-activated receptor (PPAR) agonists has been shown to induce favorable outcome after TBI in animal models^1, 4^. After the primary insult, resident microglial cells become activated, releasing cytokines^56^. Mechanical disruption of neurons and astrocytes and BBB also lead to release of pro-inflammatory cytokines and extravasation of leukocytes in the injured area^4^. Inflammatory cascades further damages potentially salvageable tissue in the perilesional area. An interesting finding in our study is that inflammatory markers were significantly decreased in the treatment group. The levels of TNF-α and IL-6 were reduced by 23.8% and 20.1%, respectively in the TNS-treatment group compared to the TBI-control animals, suggesting that TNS may have anti-inflammatory properties.

At this point, the etiology of this anti-inflammatory effect is unclear, although anti-inflammatory effect of TNS has been previously reported by Wang *et al*.^57^. In their model of epileptic rats, TNS caused reduced microglial activation as well as decreased levels of TNF-α and IL-1β. The exact mechanism of how TNS reduces inflammation has not been elucidated. However, there is evidence of interaction between the trigeminal nerve and microglia, especially in disease processes such as trigeminal neuralgia (TN)^58, 59^. In animal models, chronic constriction of the trigeminal nerve has been shown to activate microglia resulting in release of pro-inflammatory cytokines^60^. While this indicates that irritation or compression of the nerve activates microglia, it does not explain how electrical stimulation of the nerve reduces neuroinflammation. In our model, one possible explanation for this reduction in inflammation is that it is a consequence of decreased ischemia and hypoxia at the cellular level. Both ischemia and hypoxia can trigger inflammatory cascades^29, 30, 61–64^. Therefore, it is reasonable to presume that improved CBF and PbrO_2_ can dampen ischemia or hypoxia triggered inflammatory cascades. Another hypothesis is that TNS suppresses inflammation via the trigemino-vagal connection. Many studies have shown that vagus nerve stimulation has profound anti-inflammatory properties^65–68^. Furthermore, the beneficial effects of VNS in disease states such as TBI or hemorrhagic shock have been attributed to its anti-inflammatory effects^69–72^. Vagus nerve is the efferent arm of several reflex pathways that is initiated at the level of trigeminal nerve^73^. Most of these pathways have been studied and described in the context of hemodynamic changes due to trigeminal nerve stimulation in diving mammals. For example, bradycardia associated with mammalian diving reflex is due to increased vagal output to the heart, but the initiation of this reflex occurs at the level peripheral branches of trigeminal nerve, peripheral V1 branches in particular^22^. Therefore, it is reasonable to postulate that the decreases in inflammatory markers due to TNS may be mediated via this trigemino-vagal connection. Trigeminal nerve fibers project to NTS, one of the major parasympathetic centers in the brain stem. Both NTS and trigeminal sensory nuclei project to the dorsal motor nucleus (DMN) of vagus, the major parasympathetic outflow nucleus^74^. Therefore, it is essential to investigate whether TNS does in fact mediate inflammatory cascades via this pathway using vagotomized animal models.

It is worth mentioning that percutaneous TNS devices have been approved in Europe, Canada and Australia for epilepsy and depression^75, 76^. If the benefits of TNS in TBI can be replicated in large animals and ultimately in human studies, it could have tremendous impact in trauma resuscitation and TBI management. Its utility would be particularly relevant in the critical prehospital setting where percutaneous TNS could be initiated as the patient is being transported to a trauma center for a definitive care. Furthermore, there are other pathological states besides TBI where the brain is at risk for ischemic and/or inflammatory damage, such as stroke or vasospasm after subarachnoid hemorrhage. TNS could offer some benefit in these situation as well. In fact, in a recent animal study, electrical stimulation of trigeminal nerve was shown to decrease lesion volume after middle cerebral artery occlusion, and the authors attributed this to increased CBF^45^. Further studies are necessary to explore TNS’s efficacy in these pathological processes.

There are several limitations in this study. These experiments were conducted with a rat model. Results from large animal studies are needed to strengthen the conclusions of this study. In these experiments, we cannot quantify relative contribution of CBF increase between increased MAP and decreased CVR. It is reasonable to assume that the increase in CBF is a sum of these contributing sources, with decrease in the vascular resistance playing a major role, since increase in MAP was modest, and yet CBF increase was substantial. In future studies, we plan to control blood pressure parameters and measure CBF after TNS in order to determine the relative contributions. As mentioned previously, this experiment was not designed to study anti-inflammatory effects of TNS, even though the treatment group had lower levels of inflammatory markers. The exact mechanism of the observed anti-inflammatory effect is unclear, and further studies are necessary to determine the exact neural pathways involved. Another limitation of this study was that the experiments were conducted in anesthetized animals using isoflurane. Isoflurane itself can interfere with hemodynamics as it has been reported to blunt cardiac parasympathetic response^77^. Also, it has been previously reported that isoflurane can have neuroprotective properties^78^. However, these effects are accounted for since we are comparing the results to the control group which is subjected to the same experimental conditions under identical isoflurane exposure.

In this study, we have demonstrated the potential of TNS as an effective therapeutic strategy for preventing secondary injury via existing neural pathways. In summary, electrical TNS caused the significant increase in systemic blood pressure, cerebral blood flow and brain oxygen tension. Consequently, we observed that TNS following CCI attenuated some of the important consequences of TBI by reducing brain edema, blood-brain barrier permeability, lesion volume, and levels of pro-inflammatory biomarkers. These data provide strong early evidence that activation of the trigeminal nerve system affords neuroprotection following brain damage.

## Methods

### Experimental Animals

The protocol was approved by the Institutional Animal Care and Use Committee of the Feinstein Institute for Medical Research. All experiments were performed according to the approved protocol. Male Sprague-Dawley rats (325–400 g, Taconic Biosciences Inc.) were randomized three groups: (1) sham group: animals underwent surgery like other groups, but no CCI or electrical stimulation was delivered; (2) CCI-induced TBI group: similarly prepared animals received only CCI as vehicle group; (3) TNS treatment group: animals received both CCI and TNS.

### Rat Model of TBI

Controlled cortical impact (CCI) method was used to induce TBI in male Sprague-Dawley rats^79^. The head of the animal was fixed in a stereotaxic frame, and a craniotomy was performed. During surgery, anesthesia was maintained at 2.0–2.5% isoflurane and body temperature was maintained at 37 °C. CCI was delivered using electromagnetic-based device set (Impact One^TM^ Stereotaxic CCI Instrument, Leica Biosystems) at most commonly used parameters to produce severe damage: 5 mm flat impact, 3 mm depth of impact at the velocity of 6 m/s and 100 ms contact time. The “lateral” location of the impact was used: circular craniotomy of 6 mm diameter was made halfway between bregma and lambda in the parietal bone centered at 4 mm lateral from the sagittal suture. Circular bone flap was preserved in saline and replaced after the impact.

### Direct Trigeminal Nerve Stimulation

Hook electrodes (305-SL-2, PlasticsOne) were directly placed on the right and left branches of the ophthalmic nerves (V1) to provide bilateral stimulation. The ethmoidal nerves were isolated as previously described^36^. Briefly, the rats were placed in prone position. A curvilinear supraorbital incision (2–3 cm) was made, and the orbital contents were gently retracted laterally until the anterior ethmoidal nerve was visualized, passing above the inferior orbital nerve. The hook electrodes were placed directly under the nerve, ensuring adequate contact, and the electrode was connected to the electrical stimulator (Isolated Pulse Stimulator Model 2100, A-M Systems). The same procedure was repeated on the contralateral side. Stimulation parameters consist of frequency (25 Hz), intensity (1.0 V), duty cycle (1 sec on and 1 sec off), and pulse width (0.5 ms). Ophthalmic nerves were stimulated for 1 minute to investigate the effects of direct TNS on hemodynamics in naïve rats.

### Subcutaneous Trigeminal Nerve Stimulation

Electrical stimulation was performed by introducing two stainless needles (25 ga) subcutaneously bilaterally parallel to each other at imaginary lines connecting ear and eye. Rectangular cathode pulses (0.5 ms) were delivered by electrical stimulator (Isolated Pulse Stimulator Model 2100, A-M Systems). Stimulation parameters consisted of frequency (100 Hz), intensity (3.0 V), duty cycle (1 sec on and 1 sec off), and pulse width (0.5 ms). Subcutaneous TNS was delivered for 1 minute to investigate its effect on hemodynamics in naïve rats. Total 5 subcutaneous TNS (1-minute TNS in every 11 minutes) was delivered at 10 minutes after the CCI to investigate its effects on CCI-induced TBI.

### Measurement of Blood Pressure (BP)

Thin-walled polyethylene catheter (PE 50, BD Intramedic Polyethylene tubing) was placed in the left femoral artery for continuous recording of BP (SYS-BP1, World Precision Instruments, USA).

### Brain Probe Implantation and Monitoring

Animals received a 1-mm craniotomy (ML: +2--> + 3 mm; AP: −0-->1.5 mm; DV: −4.5 mm) next to 6-mm craniotomy (ML: 0--> + 6 mm; AP: −1.5-->−7.5 mm) for CCI in the same hemisphere to implant multimodal neural probe^80, 81^. One neural probe that can measure CBF, temperature and PbrO_2_ was implanted to a depth of 5 mm and fixated with dental acrylic cement. A waiting period of 60 min followed the neural probe implantation to record the brain baseline signal.

### Evaluation of Brain Edema

Brain water content, an indicator of brain edema, was measured with the wet-dry method at 24 h after CCI-induced severe TBI^82^. After the animals were killed by decapitation under deep anesthesia, their brains were removed and the ipsilateral cortical tissues were dissected and weighed immediately to get wet weight. After drying in a desiccating oven for 48 h at 100 °C, the tissues were reweighed to yield dry weight. The percentage of water in the tissues was calculated according to the formula:

$$ \% \,{\rm{brain}}\,{\rm{water}}\,=\,[({\rm{wet}}\,{\rm{weight}}\,-\,{\rm{dry}}\,{\rm{weight}})/\mathrm{wet}\,{\rm{weight}}]\,\times \,{\rm{100}}$$

### Evaluation of Blood-Brain Barrier Integrity (BBB)

BBB integrity was determined by Evans blue (EB) extravasation at 24 h after TBI^83^. Briefly, at 23 h after injury, 2% Evans blue was injected intravenously at a dose of 2 ml/kg. Animals were then re-anesthetized at 24 h and perfused with saline to remove intravascular EB dye. Animals were then decapitated, and the ipsilateral cortical tissues were dissected. Each tissue sample was weighed, homogenized in 2 ml of 50% trichloroacetic acid (w/v), and centrifuged at 10,000 rpm for 20 min. the supernatant was then diluted with solvent (one part 50% trichloroacetic acid to three parts ethanol). Tissue levels of Evans blue dye were quantitated using a spectrofluorometer at an excitation wavelength of 620 nm and an emission wavelength of 680 nm. Sample values were compared with those of Evans blue dye standards mixed with the solvent (100–1000 ng/ml).

### Quantification of Lesion Volume

Animals were euthanized under anesthesia, and then perfused transcardially with 200 ml normal saline followed by 200 ml ice-cold 4% paraformaldehyde for fixation. Brains were stored in 4% paraformaldehyde overnight at 4 °C, then transferred to a 30% sucrose solution for 3 days for cryoprotection. Brains were then sectioned coronally (40 µm) on a freezing microtome with approximately 500 µm distance between slices. Sections were mounted onto gelatin coated slides, dried overnight and stained with 0.2% cresyl violet for measurement of brain tissue loss. The stained sections were photographed using a digital pathology slide scanner (PathScan Enabler IV, Meyer Instruments, USA).

### Brain Tissue Preparation and Determination of their TNF-α and IL-6 levels

At 4 h after TBI or sham operation, the brain was rapidly harvested. Brain samples were longitudinally cut along the middle line (cortex; 7 cm). The right and left hemispheres were excised, rinsed of blood and homogenized with polytron in a homogenization buffer (phosphate-buffered saline solution, containing 0.05% Triton X-100 and protease inhibitor cocktail; pH, 7.2; 4 °C). After serval round 10 second sonication on ice, homogenates were centrifuged at 10,000 rpm for 20 min, supernatant protein concentration were measured and used for cytokine quantification. Enzyme-linked immunosorbent assay (ELISA) kits specifically for rat TNF-α and IL-6 (BD Biosciences, San Diego, CA, USA) were used. These assays were carried out according to the instructions provided by the manufacture. Brain levels of TNF-α and IL-6 were normalized to the protein concentration in the sample.

### Data Collection and Processing

All data were digitized at 2 KHz with PowerLab digitizer (Powerlab 16/SP analog/digital converter, ADInstruments). Data were stored and analyzed with LabChart 7.0 software (ADInstruments). To examine the effects of direct and subcutaneous trigeminal nerve branch stimulation on MAP, HR, PP, RR, CVR, CBF, PbrO_2_ and brain temperature, maximum/minimum values were analyzed from each TNS.

### Statistical Analysis

All data are expressed mean ± standard deviation (SD) and analyzed by SigmaStat software. The MAP, HR, PP and RR were analyzed using repeated measures ANOVA with simple contrasts when applicable. The CBF, PbrO_2_, brain temperature and CVR were similarly analyzed. The difference between multiple groups was analyzed by one-way ANOVA and post hoc test (Student-Newman-Keuls test). Student’s t-test was used when only two groups were compared. P values < 0.05 were considered significant, and P < 0.001 was considered even more significant.
